# Integrated Transcriptomic and Proteomic Analysis Reveals *Runx2b*-Associated Molecular Regulation of IB Development in *Culter alburnus*

**DOI:** 10.3390/ani16142255

**Published:** 2026-07-21

**Authors:** Yi Xu, Xiaohui Xu, Fei Li, Caiyan Li, Cancan Huang, Jianbo Zheng

**Affiliations:** 1 College of Biological and Environmental Sciences, Zhejiang Wanli University, Ningbo 315104, China; 19819983753@163.com (Y.X.); licy@zwu.edu.cn (C.L.); 2 Key Laboratory of Genetics and Breeding, Zhejiang Institute of Freshwater Fisheries, Huzhou 313001, China; lifeibest1022@163.com; 3 Yangtze Delta Region Institute of Tsinghua University, Jiaxing 314006, China; xiaohuixu71@outlook.com

**Keywords:** *Culter alburnus*, intermuscular bones, *runx2b*, gene editing, transcriptome, proteome

## Abstract

Intermuscular bones (IBs) are ossified structures within the myoseptal connective tissues of cyprinid fishes and negatively affect eating quality and processing suitability. Although *runx2b* has been shown to be essential for IB formation, the molecular changes associated with *runx2b*-mediated IB reduction or loss remain poorly understood. In this study, *runx2b*-edited *Culter alburnus* F1 individuals were generated and classified into wild-type, heterozygous mutant, and homozygous mutant groups. Skeletal staining revealed a clear genotype-associated phenotypic gradient, with normal IBs in wild-type individuals, markedly reduced IBs in heterozygous mutants, and complete IB absence in homozygous mutants. Integrated transcriptomic and proteomic analyses were then performed using matched muscle/myoseptal tissues to characterize molecular changes associated with this phenotypic gradient. Global mRNA–protein correlations were weak and DEG-DEP overlaps were limited, suggesting that transcriptomic changes alone cannot fully explain protein-level remodeling after *runx2b* mutation. Functional enrichment and integrated omics analysis highlighted coordinated changes in cytoskeletal organization, extracellular matrix remodeling, cell adhesion, calcium-related signaling, muscle-associated processes, and ossification-related pathways. Representative candidate molecules, including *MYH1s*, *CDH26*, *PLIN5*, *PAH*, *HBA*/*HBB*, *GPX4*, *KRT1*, *GSN*, *LGALS9*, and *ANXA3*, were mainly associated with structural, metabolic, and microenvironmental remodeling rather than being interpreted as direct downstream osteogenic targets of *runx2b*. Overall, these findings suggest that *runx2b*-associated IB reduction and loss may involve coordinated remodeling of the myoseptal osteogenic microenvironment rather than disruption of a single ossification pathway. This study provides a discovery-oriented multi-omics resource and a hypothesis-generating molecular model for future functional validation of IB development in cyprinid fishes.

## Simple Summary

Intermuscular bones (IBs) are small, ossified structures embedded within the muscle of many cyprinid fishes. Although they do not affect fish growth, their abundance reduces eating convenience and processing efficiency, making IB reduction an important target for aquaculture breeding. Previous studies have identified *runx2b* as a key regulator of IB development, but the molecular mechanisms underlying IB reduction or loss remain poorly understood. In this study, we used wild-type, heterozygous, and homozygous *runx2b*-edited *Culter alburnus* exhibiting normal, reduced, and absent IB phenotypes, respectively. By integrating transcriptomic and proteomic analyses, we identified molecular changes associated with progressive IB reduction and highlighted candidate genes and proteins involved in extracellular matrix remodeling, cytoskeleton organization, calcium regulation, muscle-related processes, and ossification. These findings provide new insights into the biological mechanisms of IB development and offer potential molecular targets for breeding IB-reduced or IB-free fish.

## 1. Introduction

Intermuscular bones (IBs) are a prominent skeletal feature of cyprinid fishes and develop as ossified elements within the myoseptal connective tissues [1,2]. Because abundant IBs negatively affect eating convenience and processing suitability, reducing or eliminating these structures is increasingly regarded as a promising strategy for improving product quality and breeding value in economically important cyprinids [3,4]. Accordingly, increasing attention has been paid to the developmental origin, ossification process, and molecular regulation of IBs [5,6]. Particularly in recent years, substantial progress has been made in understanding IB developmental regulation and promoting IB-targeted genetic improvement, including the identification of regulatory genes, characterization of ossification-related pathways, and generation of IB-reduced or IB-free germplasm in several aquaculture species [7,8,9].

Historically, research on teleost IBs has primarily focused on their morphological characteristics, developmental and evolutionary patterns, and ossification processes [6,10,11,12]. More recently, omics-based studies have provided new insights into IB developmental regulation by identifying multiple candidate genes associated with osteogenesis, matrix remodeling, and mineralization, including *runx2b*, *bmp6*, *scxa*, *sp7*, *alpl*, *entpd5a*, *spp1*, *bglap*, *sik1*, and *clec3bb* [13,14]. More broadly, transcriptional analyses in cultured fishes have provided useful molecular evidence for physiological regulation, immune responses, and tissue adaptation under aquaculture-related conditions [15]. Furthermore, CRISPR/Cas9-mediated knockout of *runx2b* has generated IB-free mutants in several economically important cyprinid fishes, such as amphitriploid gibel carp, blunt snout bream, and grass carp, highlighting their potential as targets for IBs genetic improvement [9,16,17]. *runx2b* is a key transcriptional regulator of osteoblast differentiation and skeletal mineralization. Its osteogenic function is closely linked to BMP, Wnt/β-catenin, Hedgehog, Notch, and TGF-β signaling pathways. These pathways regulate osteogenic commitment, extracellular matrix formation, mineralization, and bone remodeling. However, the molecular events connecting *runx2b* disruption with IB reduction or loss in teleost fishes remain unclear.

*Culter alburnus* is an economically important freshwater cyprinid widely cultured in China because of its fast growth, desirable flesh quality, and high market value [3,18]. However, the abundance of IBs substantially limits eating convenience, processing efficiency, and value-added utilization, making IB reduction an important breeding objective for this species. Here, we used *runx2b*-edited *C. alburnus* with distinct IB phenotypes to explore molecular changes associated with IB reduction and loss. We hypothesized that *runx2b* deficiency may be associated with coordinated remodeling of the IB-forming myoseptal microenvironment. Because mRNA and protein levels can differ substantially, we integrated transcriptomic and proteomic analyses to identify candidate molecular modules and gene–protein pairs. This study provides a discovery-oriented framework for future functional validation.

## 2. Materials and Methods

### 2.1. Experimental Fish and Ethics Statement

The wild-type and F0 *runx2b*-edited mutant *C. alburnus* individuals used in this study were both cultured at the experimental base of the Zhejiang Institute of Freshwater Fisheries under standard aquaculture conditions. Fish were reared in aerated tanks under a natural photoperiod at 25–28 °C, with dissolved oxygen maintained above 5.0 mg/L. They were fed a commercial diet twice daily to apparent satiation. All genotype groups were maintained under identical stocking and management conditions to minimize non-genetic effects. All animal-related experimental procedures in this study were carried out following established scientific guidelines and received approval from the Animal Ethics Committee of the Zhejiang Institute of Freshwater Fisheries to guarantee ethical compliance and animal welfare (Approval No. ZJIFF20210305, date: 3 September 2025).

### 2.2. F1 Runx2b Mutant Screening and Phenotyping

F0 *runx2b*-edited *C. alburnus* founders were generated in 2023 using CRISPR/Cas9-mediated genome editing. In the present study, sexually mature F0 individuals carrying the same mutation type were selected and paired by artificial fertilization to obtain F1 progeny. A total of 100 two-month-old F1 *C. alburnus* juveniles were randomly selected for PCR amplification and Sanger sequencing of the *runx2b* target region using specific primers (BY-*runx2b*-sRNA2-F: tgt aca cct tag ggg gtc tgt taa; BY-*runx2b*-sRNA2-R: tgc agt gtc gaa cgc gag ggg tag). Sequence alignment with the wild-type *runx2b* sequence was used to identify mutation types and classify individuals as wild-type, heterozygous mutants, or homozygous mutants. Among the 100 genotyped F1 individuals, 23 were identified as wild-type, 63 as heterozygous mutants, and 14 as homozygous mutants. Herein, 10 individuals per genotype group were selected for alizarin red skeletal staining to evaluate IB development. After staining, IBs were observed and counted under a stereomicroscope.

### 2.3. Sample Collection for Transcriptomic and Proteomic Analyses

Wild-type, heterozygous, and homozygous F1 individuals, designated as Ctrl, He, and Ho, respectively, were used for integrated transcriptomic and proteomic analyses. Based on genotyping and skeletal phenotyping, three healthy individuals with comparable body size and representative IB phenotypes were selected from each genotype group as biological replicates. Each replicate represented one independent fish rather than a pooled sample. IB-associated myoseptal/muscle tissues were collected from the trunk region between the posterior edge of the operculum and the anterior region of the caudal peduncle, mainly along the dorsal and middle myoseptal regions where IBs develop, with non-target tissues removed as much as possible. For each fish, the dissected tissue was divided for RNA and protein extraction, ensuring matched RNA-seq and proteomic analyses from the same individuals. Samples were snap-frozen in liquid nitrogen and stored at −80 °C. Because IBs are embedded within myoseptal connective tissue surrounded by muscle, these samples represented enriched IB-associated myoseptal/muscle tissues rather than purified myoseptum.

### 2.4. Transcriptome Sequencing and DEG Analysis

Total RNA was extracted from each sample and subjected to quality assessment. Qualified RNA samples were used for RNA-seq library construction and paired-end sequencing on an Illumina platform. Raw reads were filtered to remove adaptor sequences, low-quality reads, and reads containing ambiguous bases. Clean reads were aligned to the *C. alburnus* reference genome (GCA_040182925.1), and gene expression levels were quantified. DEGs were identified among Ctrl, He, and Ho groups in three pairwise comparisons—He_vs._Ctrl, Ho_vs._Ctrl, and Ho_vs._He—using Log2FC (fold change) ≥ 1.0 and adjusted *p* value < 0.01 as significance criteria.

### 2.5. Proteomic Analysis and DEP Identification

Total proteins were extracted from frozen muscle tissues of Ctrl, He, and Ho individuals using lysis buffer. After tissue grinding, sonication, and centrifugation, protein concentration was determined using the BCA method, and protein quality was assessed by SDS-PAGE. Equal amounts of peptides were dissolved in MS loading buffer and analyzed by DIA-based LC-MS/MS using a VanquishNeo LC system coupled to an Astral mass spectrometer (Thermo Fisher Scientific, Waltham, MA, USA), with chromatographic separation performed on a homemade analytical column (15 cm × 100 um, 1.7 um) using the LC gradient. DIA data were acquired in positive-ion mode using Thermo Xcalibur 4.7 and analyzed in Spectronaut v19 against the *C. alburnus* protein database using a library-free/directDIA workflow, with protein identification controlled at an FDR ≤ 1%, and protein abundance was quantified using the MaxLFQ method. The thresholds of fold change >1.2 or <0.83 and *p*-value < 0.05 were used to identify differentially expressed proteins (DEPs). Because proteome-wide multiple-testing correction was not applied to the DEP screening threshold, the DEP set was regarded as an exploratory protein-level dataset and was interpreted mainly together with pathway/module-level enrichment and transcriptomic evidence.

### 2.6. Integrated Transcriptomic and Proteomic Analysis

Transcriptome and proteome datasets were integrated using matched gene–protein pairs with available log2 fold-change values. For each comparison, Pearson and Spearman correlations were calculated to assess mRNA–protein concordance. Pearson correlation was used to evaluate the linear relationship between mRNA and protein log2 fold changes, whereas Spearman correlation was used to assess rank-based monotonic concordance, which is less sensitive to non–normal distributions and potential outliers. DEG–DEP overlaps were identified as matched pairs significantly regulated in both omics layers and classified as concordantly upregulated, concordantly downregulated, or discordant according to their regulation directions. Nine–quadrant analysis was performed by assigning each matched pair to one of nine regulatory categories based on DEG and DEP thresholds. Because transcriptomic and proteomic datasets differ in detection depth, dynamic range, and quantitative variability, omics-specific thresholds were used for DEG and DEP identification. DEG-DEP overlap was considered a conservative indicator of jointly regulated molecules, whereas nine-quadrant analysis was used to assess broader directional patterns between matched mRNA and protein changes.

### 2.7. Functional Enrichment and Candidate Gene–Protein Selection

GO and KEGG enrichment results from DEG, DEP, and integrated analyses were collected for each comparison. Enriched terms were categorized into major biological themes based on functional annotation, and representative terms were selected according to statistical significance. Candidate gene–protein pairs were further screened from the integrated dataset based on matched mRNA–protein information, concordant regulation or DEG-DEP overlap evidence, and functional relevance to the annotated biological themes.

### 2.8. Visualization of Omics Results and Network Construction

Custom Python (v3.10.12) scripts were used for data processing, statistical summarization, and visualization. Volcano plots, correlation plots, Venn diagrams, nine-quadrant plots, and bubble plots were generated to display DEGs/DEPs, mRNA–protein correlations, DEG–DEP overlaps, joint regulation patterns, and GO/KEGG enrichment results, respectively. Candidate genes/proteins were visualized using row–scaled heatmaps and paired mRNA–protein log2 fold–change plots. A putative *runx2b*–centered regulatory model was constructed in Cytoscape (v3.10.2) by linking prioritized candidates with enriched functional modules based on integrated omics evidence, functional annotation, and literature-supported biological relevance.

## 3. Results

### 3.1. Generation of Different Gene-Edited Mutant Genotypes

**Figure 1:**
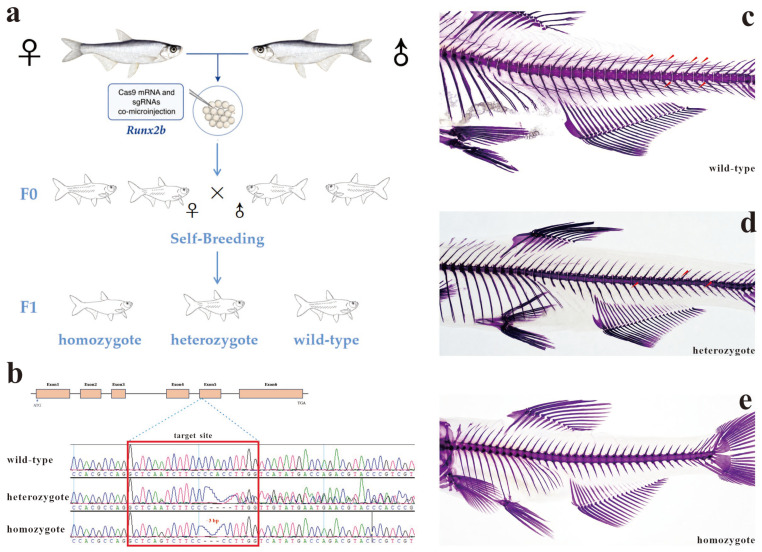
Generation and phenotypic characterization of F1 *runx2b*-edited mutants in *C. alburnus*. (**a**) Schematic workflow for generating and genotyping F1 *runx2b* mutants. (**b**) Sanger sequencing-based genotyping of F1 *runx2b* mutants in *C. alburnus*. The red box indicates the sgRNA-targeted region of the *runx2b* gene. Hyphens (-) indicate deleted nucleotides at the target site. (**c**–**e**) Alizarin red skeletal staining showing IBs phenotypes in wild-type (**c**), heterozygous (**d**), and homozygous (**e**) individuals. Red triangles indicate representative IBs.

Previously, we successfully generated *runx2b*–edited mutants in *C. alburnus* using CRISPR/Cas9-mediated targeted mutagenesis. In this study, sexually mature *runx2b*-edited *C. alburnus* individuals carrying the same mutation type were paired for breeding, and F1 progeny were successfully obtained through artificial fertilization (Figure 1a). Genotyping of 100 randomly selected two–month–old F1 individuals identified 23 wild–type individuals, 63 heterozygous mutants (*runx2b*^+/−^), and 14 homozygous mutants (*runx2b*^−/−^) (Figure 1b). Alizarin red skeletal staining revealed genotype-associated IB phenotypes: wild-type individuals had normal IB numbers (111–126, approximately 118 on average; Figure 1c), heterozygous mutants showed variably reduced IB numbers (Figure 1d), and homozygous mutants completely lacked detectable IBs (IB count = 0; Figure 1e). These results indicated that *runx2b* mutations were stably inherited in the F1 generation and that loss of *runx2b* function substantially impairs IB formation in *C. alburnus*.

### 3.2. Transcriptomic and Proteomic Profiling of Runx2b-Edited Mutants

RNA-seq data showed high sequencing quality, with 36.39–48.19 million clean reads per sample, Q30 values of 96.08–96.28%, stable GC content, and mapping rates of 92.38–93.61% to the *C. alburnus* reference genome. PCA analysis indicated partial genotype-associated separation in the transcriptomic dataset, whereas the proteomic dataset showed greater replicate–level dispersion. These results supported the reliability of subsequent DEG analysis and indicated that proteomic results should be interpreted cautiously at the pathway/module level.

**Figure 2:**
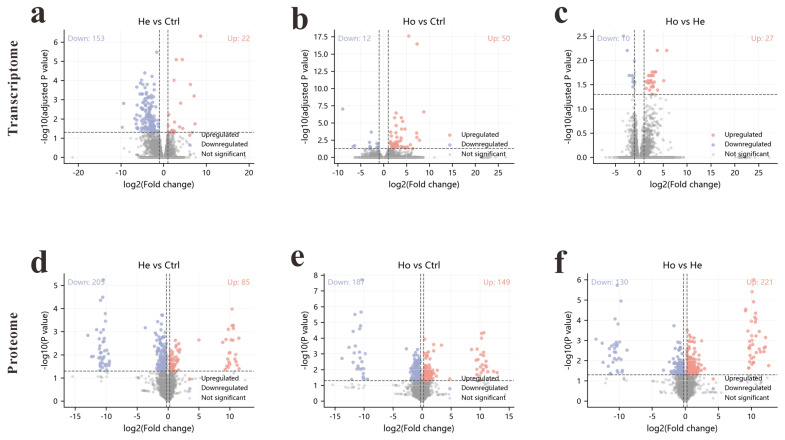
Volcano plots showing transcriptomic (**a**–**c**) and proteomic (**d**–**f**) differential expression across the three comparisons. Ctrl, wild type; He, heterozygous mutant; Ho, homozygous mutant. Dashed horizontal and vertical lines indicate the significance threshold and fold-change cutoff used for identifying differentially expressed genes/proteins.

To elucidate the molecular basis underlying *runx2b* mutation-induced IB reduction and loss, we performed integrated transcriptomic and proteomic analyses of wild–type (Ctrl), heterozygous mutant (He), and homozygous mutant (Ho) individuals. At the transcriptomic level, 175 DEGs were identified in the He group relative to Ctrl, including 22 upregulated and 153 downregulated genes (Figure 2a), whereas the Ho group showed 62 DEGs, with 50 upregulated and 12 downregulated genes (Figure 2b). Moreover, comparison between Ho and He group revealed 37 DEGs, of which 27 were upregulated and 10 were downregulated in Ho (Figure 2c). Consistently, proteomic analysis identified 290 DEPs in He_vs._Ctrl, including 85 upregulated and 205 downregulated proteins (Figure 2d), whereas Ho_vs_Ctrl showed 336 DEPs, with 149 upregulated and 187 downregulated proteins (Figure 2e). A direct Ho_vs._He comparison further revealed 351 DEPs, of which 221 were upregulated and 130 were downregulated in the Ho group (Figure 2f).

The distinct DEG and DEP profiles among the three comparisons indicated genotype-associated molecular remodeling following *runx2b* mutation. He_vs._Ctrl was characterized by more downregulated DEGs and DEPs, consistent with active molecular changes accompanying partial IB reduction, whereas Ho mutants with complete IB loss showed a distinct protein–level response. The larger number of DEPs than DEGs suggested that protein-level changes captured additional regulatory information beyond transcript abundance. Because DEPs were identified using nominal *p*–value and fold–change criteria, they were interpreted as an exploratory protein–level dataset.

### 3.3. Integrative mRNA–Protein Correlation and Nine-Quadrant Analysis

**Figure 3:**
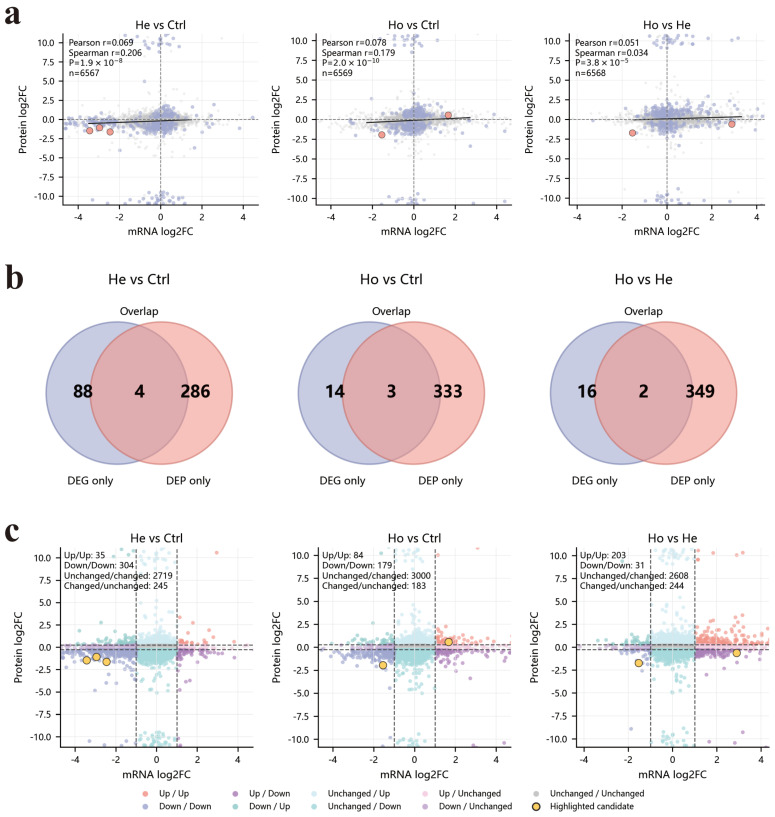
Integrative mRNA–protein correlation and nine-quadrant analysis. (**a**) Scatter plots showing the global correlation between mRNA log2FC and protein log2FC for all matched mRNA–protein pairs in each pairwise comparison. Pearson and Spearman correlation coefficients are shown in each panel. Gray points indicate non-significant pairs, blue points indicate pairs significant in one omics layer, and red points indicate DEG−DEP overlaps. (**b**) Venn diagrams showing the overlap between DEGs and DEPs in each comparison. (**c**) Nine-quadrant analysis of matched mRNA–protein pairs based on their transcriptomic and proteomic changes. The *X*-axis represents mRNA log2FC and the *Y*-axis represents protein log2FC. Colors indicate different mRNA–protein change patterns, including concordant upregulation, concordant downregulation, discordant regulation, and unchanged categories. In scatter plots (**a**,**c**), dashed vertical and horizontal lines indicate the log2 fold-change thresholds used for classification of differential expression, whereas solid lines represent linear regression fits between mRNA and protein fold changes.

To delineate the relationship between transcriptomic and proteomic alterations across different *Runx2b* genotypes, integrated mRNA–protein analysis was performed. Global mRNA–protein correlations were weak across the three comparisons, with Pearson’s correlation coefficients of 0.069, 0.078, and 0.051, respectively (Figure 3a). Consistently, DEG-DEP overlap was limited, with only 4, 3, and 2 shared genes/proteins identified in He_vs._Ctrl, Ho_vs._Ctrl, and Ho_vs._He, respectively (Figure 3b). Specifically, the He_vs_Ctrl comparison contained *MYH1s*, *MFAP4*, *perilipin6*, and *APOE*; the Ho_vs_Ctrl comparison contained *MYH1s*, *INPP5B_F*, and *SERPINB*; and the Ho_vs_He comparison contained *EVPL* and *SERPINB*. Global mRNA–protein correlations were weak across comparisons, and DEG-DEP overlaps were limited, indicating that transcript abundance alone could not fully explain protein-level remodeling. Nine-quadrant analysis further revealed comparison–specific concordant and discordant regulatory patterns. Therefore, candidate molecules were prioritized by integrating DEG–DEP overlap, concordant directional changes, pathway enrichment, functional annotation, and biological relevance to IB-associated tissue remodeling.

### 3.4. GO and KEGG Enrichment Analysis Associated with IB Development

**Figure 4:**
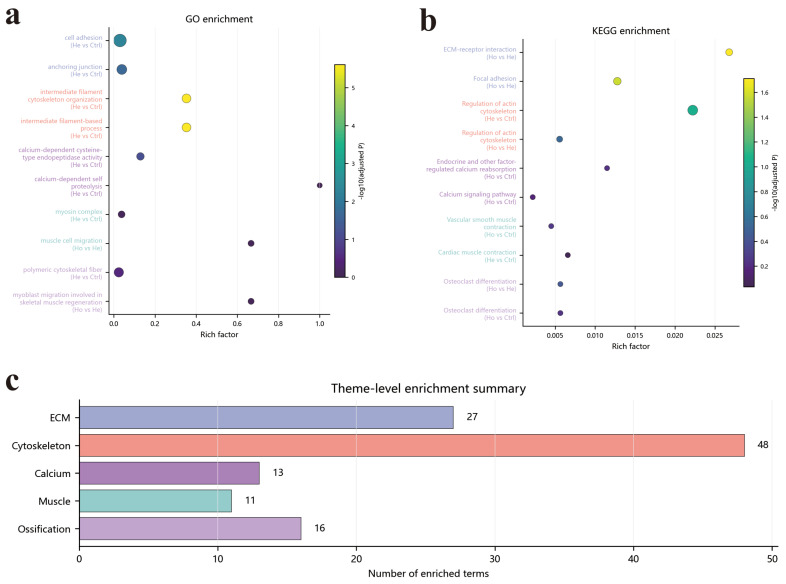
GO and KEGG enrichment analysis associated with IB development. (**a**) GO enrichment bubble plot showing representative terms related to ECM/cell adhesion, cytoskeleton, calcium signaling, muscle contraction/development, and ossification/bone mineralization. (**b**) KEGG enrichment bubble plot highlighting pathway-level enrichment related to these five biological themes, including ECM–receptor interaction, focal adhesion, calcium signaling, and muscle-related pathways. (**c**) Theme-level summary of enriched terms grouped into ECM, cytoskeleton, calcium, muscle, and ossification-related modules.

To investigate the molecular processes associated with IB development, phenotype-focused enrichment analysis was performed based on DEGs and DEPs. GO analysis highlighted terms related to cell adhesion, anchoring junction, intermediate filament organization, calcium-dependent proteolysis, myosin complex, cytoskeletal fiber, and skeletal muscle regeneration (Figure 4a). KEGG analysis identified pathways associated with ECM–receptor interaction, focal adhesion, actin cytoskeleton regulation, calcium signaling, muscle contraction, and osteoclast differentiation (Figure 4b). Cytoskeleton–related terms were the most abundant, followed by ECM/cell adhesion and ossification modules (Figure 4c). Together, these results suggest that genotype-associated IB reduction was accompanied by changes in cytoskeletal architecture, extracellular matrix organization, calcium signaling, muscle function, and osteogenic regulation in the IB-forming myoseptal region. Because these functional themes are not specific to IB development alone, they were interpreted as IB-associated tissue remodeling modules rather than direct evidence of IB-specific regulatory mechanisms.

### 3.5. Identification of Concordantly Regulated Candidate Genes/Proteins

**Figure 5:**
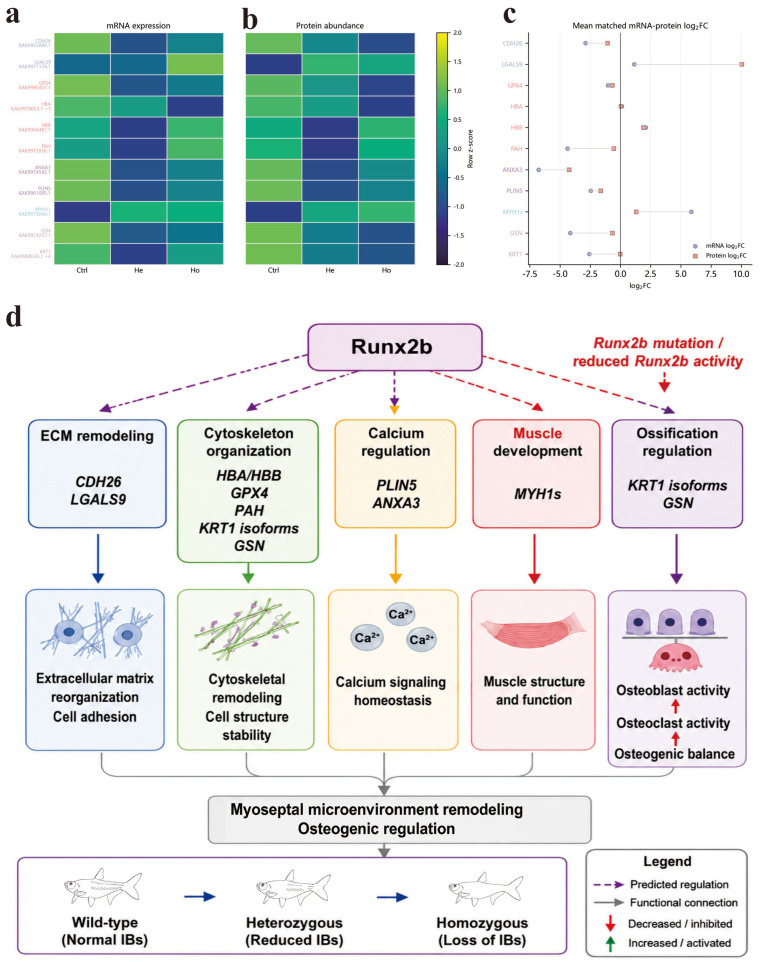
Candidate gene/protein screening and proposed *Runx2b*-centered regulatory model of IB development. (**a**) Heatmap showing row-scaled mRNA expression patterns of final candidate gene–protein pairs across Ctrl, He, and Ho groups. (**b**) Heatmap showing row–scaled protein abundance patterns for the same candidates. (**c**) Paired mRNA and protein log2FC values for each candidate. (**d**) Proposed *Runx2b*-centered regulatory model of IB development.

Representative candidate gene–protein pairs were selected based on concordant transcriptomic/proteomic regulation, functional annotation, and enrichment relevance. These candidates, including *MYH1s*, *CDH26*, *PLIN5*, *phhA*/*PAH*, *HBA*/*HBB*, *GPX4*, *KRT1*, *GSN*, *LGALS9*, and *ANXA3*, were mainly associated with ECM remodeling, cytoskeletal organization, calcium regulation, muscle–related processes, and ossification-related modules (Figure 5a,b). Because several candidates are not classical osteogenic regulators and the sampled tissues were enriched but not purified myoseptal tissues, they were interpreted as microenvironment-associated molecules rather than direct regulators of IB formation. A proposed *runx2b*-centered regulatory model was then constructed to summarize the relationships among candidate molecules, enriched functional modules, and IB-related biological processes (Figure 5d). Given the limited DEG-DEP overlap, this model should be regarded as a hypothesis-generating framework rather than a directly data-driven or experimentally validated regulatory network. The model suggests that reduced *runx2b* activity may be associated with coordinated changes in ECM remodeling, cytoskeleton organization, calcium regulation, muscle–associated processes, and ossification-related regulation. These modules may be associated with myoseptal microenvironment remodeling and altered osteogenic regulation in the IB–forming region.

## 4. Discussion

In many cyprinid fishes, IBs are ossified elements formed within the myoseptal connective tissues, and their abundance compromises eating convenience and processing suitability, making IB reduction or elimination a major goal for quality-oriented genetic improvement [11,19]. Although *runx2b* has been established as an essential regulator of IB development, the molecular cascade linking its deficiency to downstream tissue remodeling and progressive IB reduction or loss remains poorly defined [9,14,16]. In the present study, *runx2b*-edited *C. alburnus* with normal, reduced, and absent IB phenotypes provided a useful model for investigating the molecular basis of IB deficiency. Together, our integrated transcriptomic and proteomic analyses suggest that *runx2b*-associated IB reduction or loss may be associated with coordinated remodeling of the myoseptal osteogenic niche rather than disruption of a single ossification pathway [20]. These results provide association-based evidence for future functional validation.

*Runx2* is widely recognized as a master transcriptional regulator of osteoblast differentiation and skeletal mineralization, and teleost *runx2b* has been increasingly implicated in skeletal development and IB formation [21,22]. Previous gene-editing studies in zebrafish, blunt snout bream, gibel carp, and other cyprinid fishes have consistently demonstrated that disruption of *runx2b* impairs IB formation and can result in reduced or completely absent IBs, supporting the conserved and essential role of *runx2b* in teleost skeletal development [9,14,16,17]. Consistent with these previous findings, our study further confirms the conserved requirement of *runx2b* for IB formation in *C. alburnus*, as homozygous mutants completely lacked detectable IBs, whereas heterozygous mutants exhibited an intermediate reduction phenotype. This genotype–dependent phenotypic gradient, ranging from normal development to reduction and complete absence of IBs, provides a useful model for dissecting the molecular changes underlying this skeletal deficiency and represents a potential germplasm resource for IB–targeted genetic improvement [23,24]. From a breeding perspective, heterozygous mutants with reduced IB numbers may also have practical value, especially if they maintain better growth, survival, fertility, or overall fitness than homozygous IB-free mutants. Therefore, future breeding programs should evaluate both heterozygous and homozygous *runx2b*-edited fish to determine the optimal balance between IB reduction and aquaculture performance.

Because transcriptomic and proteomic data capture different regulatory layers, their integration provides a more comprehensive view of molecular remodeling associated with *runx2b* mutation [25,26]. Targeted gene-expression profiling in teleosts has also provided detailed information on the tissue- and stage-specific expression dynamics of developmental regulatory genes, thereby offering a useful complement to large-scale omics approaches [27]. A recent gene-editing and multi-omics study in grass carp similarly showed that *runx2b*–associated IB loss was accompanied by coordinated transcriptomic, proteomic, and metabolic alterations involving calcium signaling, muscle contraction, and related physiological processes [9]. Unlike transcript abundance, protein levels directly reflect the functional molecules available within cells and are additionally influenced by translational efficiency, protein stability, degradation rates, and post-translational modifications. Therefore, proteomic changes may reveal regulatory processes that cannot be inferred solely from RNA expression patterns. In this study, the weak global mRNA–protein correlations and limited DEG–DEP overlaps indicated that transcript abundance alone could not fully explain protein–level remodeling after *runx2b* mutation [28]. This discrepancy may reflect multilayer regulatory mechanisms, including post–transcriptional regulation, altered protein turnover, differential degradation, temporal delays between transcription and translation, and tissue heterogeneity [29,30]. In addition, technical factors, including differences in detection depth, dynamic range, and threshold settings between RNA-seq and proteomic analyses, may also contribute to the limited overlap. Therefore, the integrated results were interpreted mainly at the pathway and module levels, with emphasis on concordant regulatory trends and functional consistency rather than individual molecules alone.

Consistent with previous evidence linking IB formation to myoseptal development, ECM–cell adhesion, cytoskeletal regulation, and osteogenic signaling, our enrichment results suggest coordinated remodeling of the myoseptal osteogenic niche [13,31]. Recent single-cell transcriptomic and comparative genomic analyses in zebrafish further revealed the cellular heterogeneity and potential developmental origins of IB-forming cells, providing cell-level support for the importance of the local myoseptal microenvironment in IB formation [32]. ECM–receptor interaction may reflect alterations in the extracellular matrix scaffold and integrin-mediated signals required for osteogenic cell adhesion, migration, differentiation, and mineralization. Focal adhesions connect ECM receptors to the actin cytoskeleton and transmit biochemical and mechanical cues that regulate cell shape, cytoskeletal tension, and osteogenic commitment. Because IBs develop within myoseptal connective tissues exposed to muscle-generated mechanical forces, alterations in focal adhesion and actin cytoskeleton regulation may impair mechanotransduction and local osteogenic activity. PI3K/Akt-related signaling may further link integrin- and focal adhesion-associated signals with cell survival, metabolism, proliferation, and osteogenic differentiation. Consistently, developmental transcriptomic analysis in Acrossocheilus wenchowensis identified stage-dependent changes in PI3K–Akt, Wnt, MAPK, and TGF–β signaling, further supporting the involvement of dynamically coordinated osteogenic pathways in IB development [33]. Therefore, the concurrent enrichment of ECM–receptor interaction, focal adhesion, actin cytoskeleton regulation, and PI3K-related signaling may indicate remodeling of an ECM–integrin–focal adhesion–cytoskeleton axis in the IB-forming microenvironment. Accordingly, altered regulation of *CDH26* and *LGALS9* may reflect changes in adhesion and matrix interactions [34,35], whereas *KRT1* isoforms, *GSN*, and *MYH1s* may be associated with cytoskeletal organization and muscle-derived mechanical cues [36,37]. However, these enrichment results indicate biologically plausible pathway-level associations rather than direct pathway activation or direct regulation by *runx2b*.

Calcium- and metabolism–related changes may represent another important layer of myoseptal niche remodeling, given their known roles in osteogenic differentiation and IB development [38,39]. In this context, altered regulation of *ANXA3*, *PLIN5*, *GPX4*, *HBA/HBB*, and *PAH* may reflect changes in calcium-dependent membrane processes, energy metabolism, oxidative stress responses, and local tissue homeostasis [40,41,42,43,44]. However, *HBA*/*HBB*-, *PLIN5*-, *GPX4*-, and *PAH*-related changes should be interpreted cautiously, because these molecules are not classical osteogenic regulators and may reflect differences in residual blood content, vascularization, metabolism, oxidative stress, or tissue composition. Thus, they are better regarded as microenvironment-associated candidates rather than direct regulators of IB formation. Nevertheless, such changes may still affect the local physiological environment required for myoseptal ossification. Together, these findings support a putative *runx2b*-centered model linking osteogenic activity, structural organization, mechanical cues, calcium-related regulation, and metabolic homeostasis. This model remains predictive and hypothesis-generating rather than experimentally validated. These coordinated changes may be related to reduced IB formation in heterozygous mutants and IB absence in homozygous mutants.

It should be noted that the myoseptal/muscle-enriched samples used in this study inevitably contained both IB-associated connective tissue and adjacent skeletal muscle components. Therefore, the observed changes in *MYH1s*, *HBA*/*HBB*, and metabolism-related proteins should not be interpreted as evidence that these molecules directly regulate osteogenic differentiation in IB-forming cells. Instead, they may reflect alterations in local muscle organization, oxygen transport, energy metabolism, oxidative stress, or tissue homeostasis surrounding the myoseptal niche. Given that IBs develop within myoseptal connective tissues embedded in skeletal muscle, such tissue-level changes may still be biologically relevant to the IB–forming microenvironment. However, their precise cellular sources and direct functional roles require further validation.

Several limitations should be acknowledged. First, the omics analyses were based on three biological replicates per genotype, which may limit the detection of subtle molecular changes. Second, only one developmental stage and one enriched myoseptal/muscle tissue region were analyzed; therefore, early causal events cannot be distinguished from downstream consequences of IB reduction or loss. Third, because the sampled tissues were enriched but not purified myoseptal tissues, some DEGs and DEPs may originate from adjacent skeletal muscle, connective tissue, or vascular cells rather than osteogenic cells, and some muscle-, hemoglobin-, and metabolism-related changes may therefore reflect differences in tissue composition. Tissue-sampling strategy can substantially influence the downstream biological interpretation of sequencing-based datasets and should be carefully considered when evaluating tissue-level molecular changes [45]. Future studies combining laser capture microdissection, spatial transcriptomics, single-cell sequencing, and cell–type–specific validation will be required to resolve the cellular origins of these molecular changes.

## 5. Conclusions

In conclusion, this study provides an integrated transcriptomic and proteomic view of *runx2b*-associated IB deficiency in *C. alburnus*. The results support a putative model in which *runx2b* mutation is associated with coordinated remodeling of the myoseptal osteogenic niche, involving ECM organization, cytoskeletal architecture, calcium-related regulation, muscle-associated cues, metabolic homeostasis, and ossification balance. Although the proposed candidate molecules and regulatory relationships require further functional validation, these findings broaden our understanding of IB development from a single osteogenic process to a coordinated, multi-module tissue-remodeling framework. These findings provide new insights into *runx2b*-associated skeletal development and offer a valuable molecular resource for future functional genomics studies, selective breeding, and genome-editing strategies aimed at reducing intermuscular bones in *Culter alburnus*.

## Data Availability

The processed source data supporting Figures have been deposited in Figshare at https://doi.org/10.6084/m9.figshare.33044435. RNA-seq data are available from the NCBI Gene Expression Omnibus under accession number GSE337778. DIA-based LC-MS/MS proteomics data are available from ProteomeXchange/PRIDE under accession number PXD080591. Custom scripts used for data analysis are available on GitHub at https://github.com/shefferxu/runx2b-ib-omics (accessed on 16 July 2026).
